# Planetary health diet index and mortality among US cancer survivors: mediating roles of systemic immune-inflammation index and neutrophil-to-lymphocyte ratio

**DOI:** 10.1186/s12937-025-01097-6

**Published:** 2025-02-22

**Authors:** Haolin Chen, Qinglong Yang, Huihui Zheng, Jianhui Tan, Jiayi Xie, Miaojie Xu, Xue Ouyang, Zhiyang Li, Yexi Chen

**Affiliations:** 1 Department of Thyroid, Breast and Hernia Surgery, General Surgery, The Second Affiliated Hospital of Shantou University Medical College, Shantou, 515000 Guangdong China; 2 Department of Urology, The Second Affiliated Hospital of Shantou University Medical College, Shantou, 515000 Guangdong China

**Keywords:** Planetary Health Diet, Mortality, Cancer, Systemic immune-inflammation index, Neutrophil-to-lymphocyte ratio

## Abstract

**Background:**

Cancer-related deaths and environmental issues pose significant global challenges. The Planetary Health Diet (PHD) is a healthy dietary pattern that simultaneously promotes human health and ecology. This study aims to investigate the association between the Planetary Health Diet Index (PHDI) and mortality among cancer survivors, as well as the mediating role of inflammation between PHDI and all-cause mortality.

**Methods:**

This study analyzed data from 3,442 cancer survivors enrolled in the United States National Health and Nutrition Examination Survey between 1999 and 2018. To investigate the association between PHDI and mortality, we applied weighted multivariate Cox proportional hazards regression, restricted cubic spline analysis, subgroup analysis, and sensitivity analysis. The mediating effects of the Systemic Immune-Inflammation Index (SII) and Neutrophil-to-Lymphocyte Ratio (NLR) were assessed using the bootstrap method with 1000 simulations.

**Results:**

In the fully adjusted model, each 10-point PHDI increase correlated with a 9% decrease in all-cause mortality (HR, 0.91; 95% CI, 0.86–0.95), a 10% decrease in cancer mortality (HR, 0.90; 95% CI, 0.83–0.99), and a 10% decrease in non-cancer mortality (HR, 0.90; 95% CI, 0.85–0.96). The PHDI was significantly inversely correlated with SII and NLR, which were positively related to all-cause mortality. The mediation proportions of SII and NLR between the PHDI and all-cause mortality were 6.52% and 8.52%, respectively.

**Conclusions:**

Adherence to the PHD is associated with reduced all-cause, cancer, and non-cancer mortality among cancer survivors. Additionally, SII and NLR may mediate the relationship between PHDI and all-cause mortality.

**Supplementary Information:**

The online version contains supplementary material available at 10.1186/s12937-025-01097-6.

## Introduction

Cancer remains a major global public health issue. According to the latest data from the International Agency for Research on Cancer, 2022 saw 20 million new cancer cases and 9.7 million cancer-related deaths worldwide [1]. It is projected that by 2050, the annual incidence of cancer will increase by 77% compared to 2022, surpassing 35 million new cases [1]. Cancer not only inflicts profound physical harm and diminishes the quality of life for patients but also exacts a significant economic toll due to costly treatment regimens. Despite remarkable progress in diagnostic and therapeutic modalities, cancer continues to be a common cause of death. In the United States alone, an estimated 611,720 individuals are projected to succumb to cancer in 2024, equating to approximately 1,680 deaths per day [2]. Given these alarming data, it is of great significance to investigate potential strategies for reducing mortality in cancer survivors.

Mortality in cancer survivors is a multifactorial outcome, influenced by genetic predisposition, age, weight, viral infections, ultraviolet exposure, alcohol consumption, smoking, exercise, and dietary habits [3]. Diet, being a modifiable factor, has been studied for its potential to mitigate mortality risk, with consumption of vegetables, fruits, legumes, and whole grains showing promise [4, 5]. The associations between various dietary patterns and cancer prognosis have been investigated, including the Mediterranean diet [6], the prudent/healthy dietary pattern [7], and the Dietary Guidelines for Americans [8]. However, these patterns focus primarily on diet quality and adherence to specific health guidelines, without considering environmental sustainability. In response to global sustainability concerns and to improve human health, the EAT-Lancet Commission introduced the Planetary Health Diet (PHD) in 2019 [9]. This diet recommends a daily intake of 2,500 kcal, prioritizes plant-based foods, and restricts red and processed meats [9]. The Planetary Health Diet Index (PHDI) quantitatively assesses adherence to this diet across 14 food groups, with higher scores reflecting greater compliance [10].

Compared to other dietary patterns, the PHD offers significant advantages in preventing severe environmental degradation. Previous studies have shown that the PHD has the potential to reduce freshwater use, greenhouse gas footprint, and global agricultural land use while protecting biodiversity [11–13]. Widespread adoption of this dietary pattern is projected to put the food system on a sustainable track by 2050 [14]. In addition, studies have correlated strict adherence to the PHD with diminished all-cause and disease-specific mortalities, including those from cancer, cardiovascular diseases, respiratory diseases, and neurodegenerative conditions [15, 16]. Therefore, the PHD is distinctive for its dual benefit of ecological sustainability and human health, offering a holistic framework for advancing both environmental and public health goals. However, the PHD’s influence on cancer survivors is less explored, and its mortality risk reduction potential in this cohort is not well-established. To fill this research gap, we carried out the first large-scale prospective cohort study to explore the relationship between the PHDI and mortality in cancer survivors. This survey identifies a dietary pattern that promotes health and environmental conservation, which is crucial for global dietary policy formulation and cancer survivor management.

In addition, inflammation’s role in the tumor microenvironment is increasingly recognized for its facilitation of angiogenesis and cancer cell proliferation, which are closely related to the pathogenesis and progression of tumors [17]. The Systemic Immune-Inflammation Index (SII) and Neutrophil-to-Lymphocyte Ratio (NLR) serve as indicators of systemic inflammatory responses and have been closely linked to cancer prognosis. Specifically, higher SII values have been correlated with adverse outcomes in colorectal cancer, hepatocellular carcinoma, and prostate cancer patients [18–20]. Similarly, an increased NLR has been related to reduced overall survival in individuals diagnosed with nasopharyngeal carcinoma, prostate cancer, endometrial cancer, and breast cancer [21]. Additionally, consumption of plant-based foods has been studied to be associated with reduced levels of inflammation [22, 23]. Given that the PHD predominantly consists of plant-based foods, this study hypothesizes that inflammation may act as a mediating factor between the PHD and all-cause mortality among cancer survivors.

In this study, we aim to examine the association between PHDI and all-cause, cancer, and non-cancer mortality among cancer survivors, and to explore the potential mediating role of inflammatory markers (SII and NLR) in the relationship between PHDI and all-cause mortality.

## Materials and methods

### Study population

**Fig. 1:**
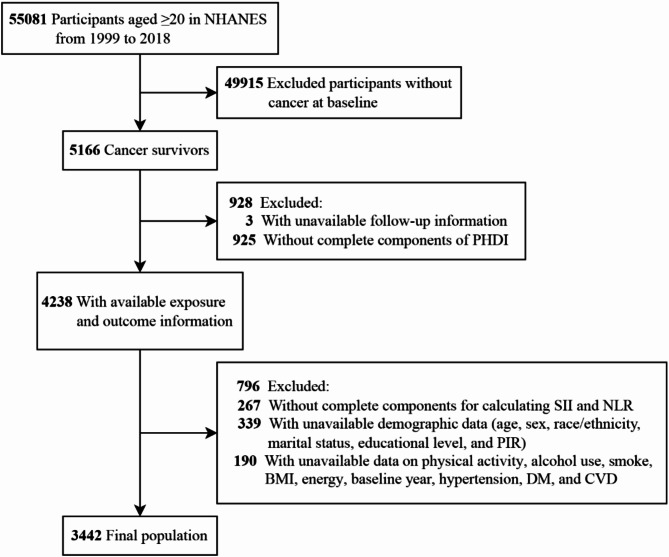
The flow chart of the study. Abbreviations: BMI, body mass index; CVD, cardiovascular disease; DM, diabetes mellitus; NHANES, National Health and Nutrition Examination Survey; NLR, neutrophil-to-lymphocyte ratio; PHDI, Planetary Health Diet Index; PIR, poverty income ratio; SII, systemic immune-inflammation index

This study leveraged data from 10 survey cycles of the National Health and Nutrition Examination Survey (NHANES), which employed a complex, multi-stage, clustered probability design comprising several stages of stratification. The National Center for Health Statistics (NCHS) Research Ethics Review Board has approved the protocol, and all participants have provided informed written consent at the time of enrollment. The sample initially encompassed 55,081 individuals aged 20 and above. We then established a series of exclusion criteria. Firstly, we excluded 49,915 participants without cancer at baseline. Subsequently, we further excluded 3 individuals with unavailable follow-up information, 925 without complete components of PHDI, 267 with missing SII and NLR measurement data, 339 with missing demographic data, and 190 individuals with missing data on lifestyle and health status-related variables. Ultimately, our study was conducted with the involvement of 3,442 cancer survivors (Fig. 1).

### Diagnosis of cancer

Cancer survivors referred to participants who responded in the affirmative to the inquiry “Have you ever been told by a doctor or other health professional that you had cancer or a malignancy of any kind?” [24]. Cancer types were then classified into nine categories, as detailed in the Supplementary Method.

### Measurement of the PHDI

The PHDI quantifies the congruence of dietary habits with the PHD. This metric is a scientifically grounded tool to foster health and environmental sustainability in dietary practices. It was calculated using self-reported dietary data and referenced to the recommended ranges delineated in the scientific report of the EAT-Lancet committee [10, 11]. As shown in Supplementary Table 1, the PHDI consists of 14 food groups, six of which are considered adequacy components and encouraged to be consumed, while eight are moderation components and recommended to be consumed in a restricted manner. The scores for each group are on a scale of 0 to 10, with the PHDI’s total score being the aggregate of these individual scores, thus spanning a potential range from 0 to 140.

### Definition of SII and NLR

Neutrophil, lymphocyte, and platelet counts are assessed using the Beckman Coulter MAXM or Beckman Coulter HMX, with detailed laboratory methodologies available on the NHANES website. SII = (neutrophils × platelets)/lymphocytes [18], NLR = neutrophils/lymphocytes [21].

### Ascertainment of mortality

Mortality data were sourced from the National Death Index death certificate records, accessed via the NCHS database. The ascertainment of causes of death adhered to the criteria outlined in the International Classification of Diseases, Tenth Revision (ICD-10). All-cause mortality denoted deaths attributed to any cause, while cancer mortality specifically referred to deaths attributed to malignant neoplasms, with ICD-10 codes ranging from C00 to C97. This study followed participants from their baseline interview date until the date of their death or the study cut-off date, which was December 31, 2019.

### Covariates

In this study, covariates were identified according to three criteria: clinical relevance, univariate regression *P*-value less than 0.05, and sufficient sample data. The ascertainment of clinical relevance was through clinical experience, previous literature [8, 15], and directed acyclic graphs (Supplementary Fig. 1). Covariates included age, sex, race/ethnicity, marital status, educational attainment, poverty income ratio (PIR), physical activity, alcohol use, smoking, Body Mass Index (BMI), energy, baseline year, hypertension, diabetes mellitus (DM), and cardiovascular disease (CVD). For detailed definitions and categorizations of the covariates, please refer to the Supplementary Method.

### Statistical analysis

We accounted for the complex sampling design and applied appropriate weights following the NHANES Analytic Guidelines. Continuous variables were analyzed via the Wilcoxon rank-sum test for complex survey samples, while categorical variables were analyzed via the chi-square test with Rao & Scott second-order correction. Participant characteristics were analyzed by PHDI quintiles, with continuous variables presented as means and standard errors (SE) and categorical variables as counts and percentages (%).

PHDI was explored as both a continuous variable (per 10-point increment) and a categorical variable (quintiles). In regression analyses, we natural log-transformed SII and NLR to normalize their distributions. The relationships between PHDI and all-cause, cancer, and non-cancer mortality and between SII, NLR, and all-cause mortality were analyzed using weighted multivariate Cox proportional hazards regression models. We employed weighted multiple linear regression analysis models to explore the relationship between PHDI and SII and NLR. We adjusted for various potential confounders: Model 1 adjusted for age. Model 2 adjusted for age, sex, race/ethnicity, marital status, educational attainment, PIR, physical activity, alcohol consumption, smoking, BMI, energy, and baseline year. Model 3 further adjusted for hypertension, DM, and CVD on top of Model 2. Restricted cubic spline (RCS) analysis with three knots was employed to assess potential non-linear associations between PHDI and all-cause, cancer, and non-cancer mortality.

To ascertain whether SII and NLR act as mediators in the relationship between PHDI and all-cause mortality, we utilized the R package “mediation” and performed 1000 bootstrap simulations to estimate the mediation effects of each mediator and calculate the proportion of mediation. The direct effect (DE) denotes the consequence of PHDI on all-cause mortality without mediation effects, while the indirect effect (IE) signifies the effect of PHDI on all-cause mortality through mediators. The mediation proportion was calculated by dividing the IE by the total effect (TE). We conducted subgroup analyses, stratified by confounding factors, and evaluated interactions. We also examined the associations between PHDI components and mortality, as well as the relationship between the adjusted PHDI score (excluding each component) and mortality. Additionally, we explored the associations between PHDI and mortality across different cancer types. To mitigate the potential for reverse causality, sensitivity analyses were conducted by excluding participants who were deceased within 24 months. Additionally, missing data for covariates were handled through the utilization of multiple imputations with chained equations. All statistical analyses were performed with R (version 4.3.1) and a two-sided *P*-value less than 0.05 was deemed statistically significant.

## Results

### Baseline characteristics

Table 1 presents the baseline characteristics of 3,442 participants stratified by quintiles of the PHDI. The participants’ weighted mean age (SE) was 62.45 (0.33) years, and 57.49% were female. Compared to participants in the lower PHDI quintiles, those in the higher quintiles tended to be older, female, married, educated beyond high school, have sufficient physical activity, have mild alcohol consumption, never smoke, have a higher PIR, and have a lower BMI, energy intake, and SII.

**Table 1:** Baseline characteristics of US cancer survivors and stratified by quintile of PHDI, NHANES 1999 to 2018

	PHDI quintile						
Characteristic	All (3,442)	Q1 (689)	Q2 (688)	Q3 (688)	Q4 (688)	Q5 (689)	*P* value
Weighted population	15,423,854	2,891,774	3,105,485	2,934,917	3,120,727	3,370,951	
Age, Mean (SE), years	62.45 (0.33)	58.21 (0.74)	62.04 (0.72)	63.29 (0.68)	64.14 (0.61)	64.15 (0.63)	< 0.001
Age group, No. (%)							< 0.001
20–64	1,347 (50.05)	338 (59.17)	300 (53.55)	242 (46.32)	235 (46.17)	232 (45.85)	
≥ 65	2,095 (49.95)	351 (40.83)	388 (46.45)	446 (53.68)	453 (53.83)	457 (54.15)	
Sex, No. (%)							< 0.001
Female	1,793 (57.49)	294 (46.85)	357 (58.49)	357 (57.81)	395 (61.44)	390 (61.77)	
Male	1,649 (42.51)	395 (53.15)	331 (41.51)	331 (42.19)	293 (38.56)	299 (38.23)	
Race/ethnicity, No. (%)							0.02
Mexican American	221 (1.97)	49 (2.41)	44 (1.80)	39 (1.59)	41 (1.74)	48 (2.29)	
Non-Hispanic Black	425 (4.55)	124 (7.04)	88 (4.94)	86 (4.84)	78 (3.87)	49 (2.41)	
Non-Hispanic White	2,510 (88.47)	459 (83.89)	505 (88.43)	505 (89.35)	511 (89.68)	530 (90.55)	
Other Hispanic	156 (2.08)	31 (3.06)	32 (1.80)	36 (2.22)	34 (1.82)	23 (1.62)	
Other Race	130 (2.93)	26 (3.77)	19 (3.02)	22 (1.88)	24 (2.97)	39 (3.14)	
Marital status, No. (%)							< 0.001
Married	2,052 (64.29)	386 (59.07)	408 (62.97)	406 (62.82)	419 (66.80)	433 (68.94)	
Never married	186 (4.80)	53 (7.77)	52 (6.63)	34 (4.10)	24 (3.15)	23 (2.72)	
Living with a partner	97 (3.06)	31 (5.40)	18 (3.28)	23 (3.53)	17 (2.43)	8 (1.02)	
Other	1,107 (27.85)	219 (27.76)	210 (27.12)	225 (29.55)	228 (27.63)	225 (27.31)	
Educational attainment, No. (%)							< 0.001
Less than high school	717 (13.20)	187 (17.92)	163 (14.97)	142 (13.58)	134 (12.75)	91 (7.58)	
High school or equivalent	793 (22.49)	181 (27.44)	185 (27.80)	161 (21.84)	138 (18.81)	128 (17.33)	
Above high school	1,932 (64.31)	321 (54.64)	340 (57.23)	385 (64.58)	416 (68.43)	470 (75.09)	
Baseline year^a^, No. (%)							0.002
1999–2000	223 (5.67)	58 (7.60)	55 (7.49)	45 (6.45)	37 (4.37)	28 (2.84)	
2001–2002	333 (9.45)	69 (11.09)	77 (10.91)	67 (8.95)	52 (6.89)	68 (9.49)	
2003–2004	338 (9.25)	70 (10.26)	88 (12.17)	55 (8.10)	81 (11.35)	44 (4.73)	
2005–2006	285 (8.29)	65 (9.18)	54 (8.01)	66 (10.14)	51 (6.94)	49 (7.43)	
2007–2008	397 (9.21)	79 (10.85)	67 (7.58)	93 (10.36)	83 (8.80)	75 (8.68)	
2009–2010	428 (10.48)	82 (9.07)	89 (10.14)	72 (9.69)	92 (11.40)	93 (11.86)	
2011–2012	304 (10.05)	55 (8.83)	47 (5.66)	63 (10.48)	67 (12.92)	72 (12.11)	
2013–2014	390 (12.80)	68 (10.46)	60 (10.68)	73 (12.48)	92 (13.80)	97 (16.10)	
2015–2016	375 (12.90)	72 (12.75)	74 (12.86)	84 (14.48)	64 (11.48)	81 (13.00)	
2017–2018	369 (11.91)	71 (9.90)	77 (14.50)	70 (8.87)	69 (12.06)	82 (13.76)	
PIR, Mean (SE)	3.29 (0.04)	2.96 (0.08)	3.19 (0.07)	3.19 (0.08)	3.42 (0.07)	3.65 (0.07)	< 0.001
Physical activity, min/wk, No. (%)							< 0.001
None (inactive)	1,155 (28.61)	259 (31.24)	245 (32.43)	241 (29.68)	225 (28.02)	185 (22.47)	
0 to < 150 (insufficiently active)	722 (21.72)	152 (24.98)	153 (23.28)	135 (21.14)	147 (22.88)	135 (16.93)	
≥ 150 (active)	1,565 (49.67)	278 (43.78)	290 (44.29)	312 (49.18)	316 (49.10)	369 (60.60)	
Alcohol use, No. (%)							0.001
Never	422 (9.69)	59 (6.49)	101 (12.10)	91 (10.53)	78 (8.03)	93 (11.04)	
Former	931 (22.27)	216 (26.15)	187 (23.29)	193 (24.53)	194 (22.82)	141 (15.55)	
Mild	1,408 (43.25)	245 (39.07)	248 (38.19)	278 (41.21)	297 (45.46)	340 (51.20)	
Moderate	375 (14.25)	75 (13.74)	89 (15.17)	72 (14.28)	64 (12.81)	75 (15.13)	
Heavy	306 (10.54)	94 (14.56)	63 (11.25)	54 (9.45)	55 (10.88)	40 (7.08)	
Smoke, No. (%)							< 0.001
Never	1,510 (44.77)	234 (35.70)	285 (43.72)	309 (47.15)	322 (43.81)	360 (52.32)	
Former	1,432 (39.56)	280 (39.41)	278 (36.09)	285 (36.72)	299 (43.18)	290 (41.99)	
Now	500 (15.68)	175 (24.89)	125 (20.19)	94 (16.13)	67 (13.01)	39 (5.69)	
BMI, mean (SE), kg/m^2^	28.88 (0.14)	29.78 (0.32)	28.94 (0.35)	28.80 (0.36)	29.17 (0.33)	27.87 (0.31)	0.01
Energy, mean (SE), kcal/d	1,957.15 (16.53)	2,200.44 (40.56)	1,962.55 (36.97)	1,903.07 (40.44)	1,896.95 (28.35)	1,846.29 (33.51)	< 0.001
Hypertension, No. (%)							0.045
No	1,229 (41.88)	274 (46.45)	227 (38.92)	237 (40.22)	223 (37.93)	268 (45.81)	
Yes	2,213 (58.12)	415 (53.55)	461 (61.08)	451 (59.78)	465 (62.07)	421 (54.19)	
DM, No. (%)							0.21
No	2,558 (78.76)	531 (79.24)	527 (81.41)	496 (77.92)	498 (75.23)	506 (79.91)	
Yes	884 (21.24)	158 (20.76)	161 (18.59)	192 (22.08)	190 (24.77)	183 (20.09)	
CVD, No. (%)							0.66
No	2,594 (80.42)	523 (80.47)	510 (80.70)	510 (80.44)	513 (78.35)	538 (82.04)	
Yes	848 (19.58)	166 (19.53)	178 (19.30)	178 (19.56)	175 (21.65)	151 (17.96)	
SII, Mean (SE)	596.41 (7.88)	620.82 (17.52)	609.30 (18.57)	609.88 (16.61)	607.72 (16.08)	541.41 (15.91)	0.01
NLR, Mean (SE)	2.47 (0.03)	2.53 (0.06)	2.49 (0.07)	2.54 (0.08)	2.52 (0.05)	2.32 (0.06)	0.053

Abbreviations: BMI, body mass index; CVD, cardiovascular disease; DM, diabetes mellitus; NHANES, National Health and Nutrition Examination Survey; NLR, neutrophil-to-lymphocyte ratio; PHDI, Planetary Health Diet Index; PIR, poverty income ratio; Q, quintile; SII, systemic immune-inflammation index; SE, standard error. All means and SEs for continuous variables and percentages for categorical variables were weighted

^a^. Refers to the year participants attended NHANES

### Association between PHDI with all-cause, cancer, and noncancer mortality

During a median follow-up of 10.4 years, 1,191 all-cause, 363 cancer, and 828 non-cancer deaths occurred. Table 2 demonstrates the relationship between PHDI and all-cause, cancer, and non-cancer mortality. In Model 3, for each 10-point rise in PHDI, there is a 9% decline in all-cause mortality (HR, 0.91; 95% CI, 0.86–0.95), a 10% decline in cancer mortality (HR, 0.90; 95% CI, 0.83–0.99), and a 10% decline in non-cancer mortality (HR, 0.90; 95% CI, 0.85–0.96). Furthermore, in Model 3, compared with the bottom PHDI quintile, participants in the top quintile experienced a 33% decreased risk of all-cause mortality (HR, 0.67; 95% CI, 0.53–0.83) and a 38% reduced risk of non-cancer mortality (HR, 0.62; 95% CI, 0.47–0.81).

**Table 2:** Association of PHDI with all-cause, cancer, and noncancer mortality among US cancer survivors

			Model 1^b^		Model 2^c^		Model 3^d^	
Mortality Outcome	Death/No.	Weighted death(%)^a^	HR(95%CI)	*P* value	HR(95%CI)	*P* value	HR(95%CI)	*P* value
**All-Cause Mortality**								
PHDI score^e^			0.84(0.80, 0.89)	< 0.001	0.91(0.86, 0.95)	< 0.001	0.91(0.86, 0.95)	< 0.001
Quintile								
Q1	254/689	798,255(27.60%)	1[Reference]		1[Reference]		1[Reference]	
Q2	263/688	839,150(27.02%)	0.78(0.64, 0.95)	0.02	0.87(0.72, 1.06)	0.17	0.85(0.69, 1.04)	0.11
Q3	260/688	852,952(29.06%)	0.81(0.66, 0.99)	0.04	0.96(0.79, 1.18)	0.73	0.95(0.77, 1.17)	0.62
Q4	225/688	755,572(24.21%)	0.65(0.52, 0.81)	< 0.001	0.82(0.66, 1.01)	0.06	0.79(0.63, 0.98)	0.03
Q5	189/689	647,376(19.20%)	0.50(0.39, 0.63)	< 0.001	0.67(0.54, 0.85)	< 0.001	0.67(0.53, 0.83)	< 0.001
Trend test				< 0.001		0.001		< 0.001
**Cancer Mortality**								
PHDI score^e^			0.82(0.75, 0.90)	< 0.001	0.91(0.83, 0.99)	0.02	0.90(0.83, 0.99)	0.02
Quintile								
Q1	85/689	260,929(9.02%)	1[Reference]		1[Reference]		1[Reference]	
Q2	81/688	231,440(7.45%)	0.71(0.50, 1.02)	0.07	0.84(0.57, 1.23)	0.36	0.85(0.57, 1.25)	0.40
Q3	79/688	264,044(9.00%)	0.83(0.57, 1.19)	0.31	1.05(0.74, 1.49)	0.79	1.05(0.74, 1.50)	0.78
Q4	65/688	217,125(6.96%)	0.62(0.41, 0.96)	0.03	0.84(0.57, 1.24)	0.39	0.85(0.57, 1.27)	0.42
Q5	53/689	202,065(5.99%)	0.53(0.35, 0.80)	0.003	0.79(0.52, 1.19)	0.26	0.79(0.52, 1.20)	0.27
Trend test				0.003		0.31		0.32
**Noncancer Mortality**								
PHDI score^e^			0.85(0.81, 0.90)	< 0.001	0.91(0.86, 0.96)	< 0.001	0.90(0.85, 0.96)	< 0.001
Quintile								
Q1	169/689	537,326(18.58%)	1[Reference]		1[Reference]		1[Reference]	
Q2	182/688	607,710(19.57%)	0.81(0.63, 1.04)	0.10	0.89(0.70, 1.13)	0.33	0.85(0.66, 1.09)	0.20
Q3	181/688	588,908(20.07%)	0.80(0.63, 1.03)	0.08	0.94(0.71, 1.22)	0.63	0.91(0.70, 1.19)	0.49
Q4	160/688	538,447(17.25%)	0.66(0.51, 0.87)	0.003	0.81(0.61, 1.08)	0.15	0.77(0.57, 1.03)	0.08
Q5	136/689	445,311 (13.21%)	0.49(0.37, 0.64)	< 0.001	0.63(0.49, 0.83)	< 0.001	0.62(0.47, 0.81)	< 0.001
Trend test				< 0.001		< 0.001		< 0.001

Abbreviations: BMI, body mass index; CVD, cardiovascular disease; DM, diabetes mellitus; PHDI, Planetary Health Diet Index; PIR, poverty income ratio; Q, quintile; HR, Hazard Ratio; CI, Confidence interval

^a^. Weighted number and proportion of deaths by PHDI quintile

^b^. Adjusted for age

^c^. Adjusted for age, sex, race/ethnicity, marital status, educational level, PIR, physical activity, alcohol use, smoke, BMI, energy, baseline year

^d^. Adjusted for age, sex, race/ethnicity, marital status, educational level, PIR, physical activity, alcohol use, smoke, BMI, energy, baseline year, hypertension, DM, and CVD

^e^. PHDI score was entered as a continuous variable per 10 points increase

**Fig. 2:**
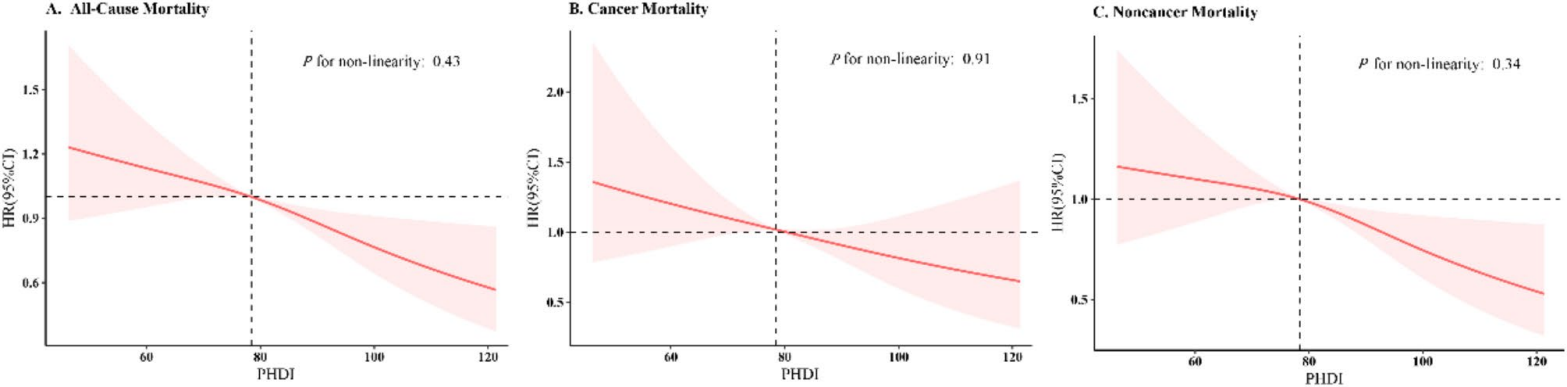
Association of PHDI with all-cause, cancer, and noncancer mortality among US cancer survivors using restricted cubic splines regression. Note: **(A)** Association between PHDI and all-cause mortality among US cancer survivors. **(B)** Association between PHDI and cancer mortality among US cancer survivors. **(C)** Association between PHDI and noncancer mortality among US cancer survivors. Abbreviations: BMI, body mass index; CVD, cardiovascular disease; DM, diabetes mellitus; PHDI, Planetary Health Diet Index; PIR, poverty income ratio; HR, Hazard Ratio; CI, Confidence interval. The model was adjusted for age, sex, race/ethnicity, marital status, educational level, PIR, physical activity, alcohol use, smoke, BMI, energy, baseline year, hypertension, DM, and CVD

The RCS results in Fig. 2 indicate a linear relationship between PHDI and all-cause mortality, as well as between PHDI and non-cancer mortality. Supplementary Table 2 highlights significant relationships between specific PHDI components and all-cause mortality, including scores for whole fruits, non-starchy vegetables, nuts and seeds, unsaturated oils, saturated oils and trans fats, and added sugar and fruit juice. Supplementary Table 3 presents the association between the adjusted PHDI score (excluding each component) and mortality. Supplementary Table 4 details the relationship between PHDI and mortality across different cancer types.

### Mediation analyses

**Fig. 3:**
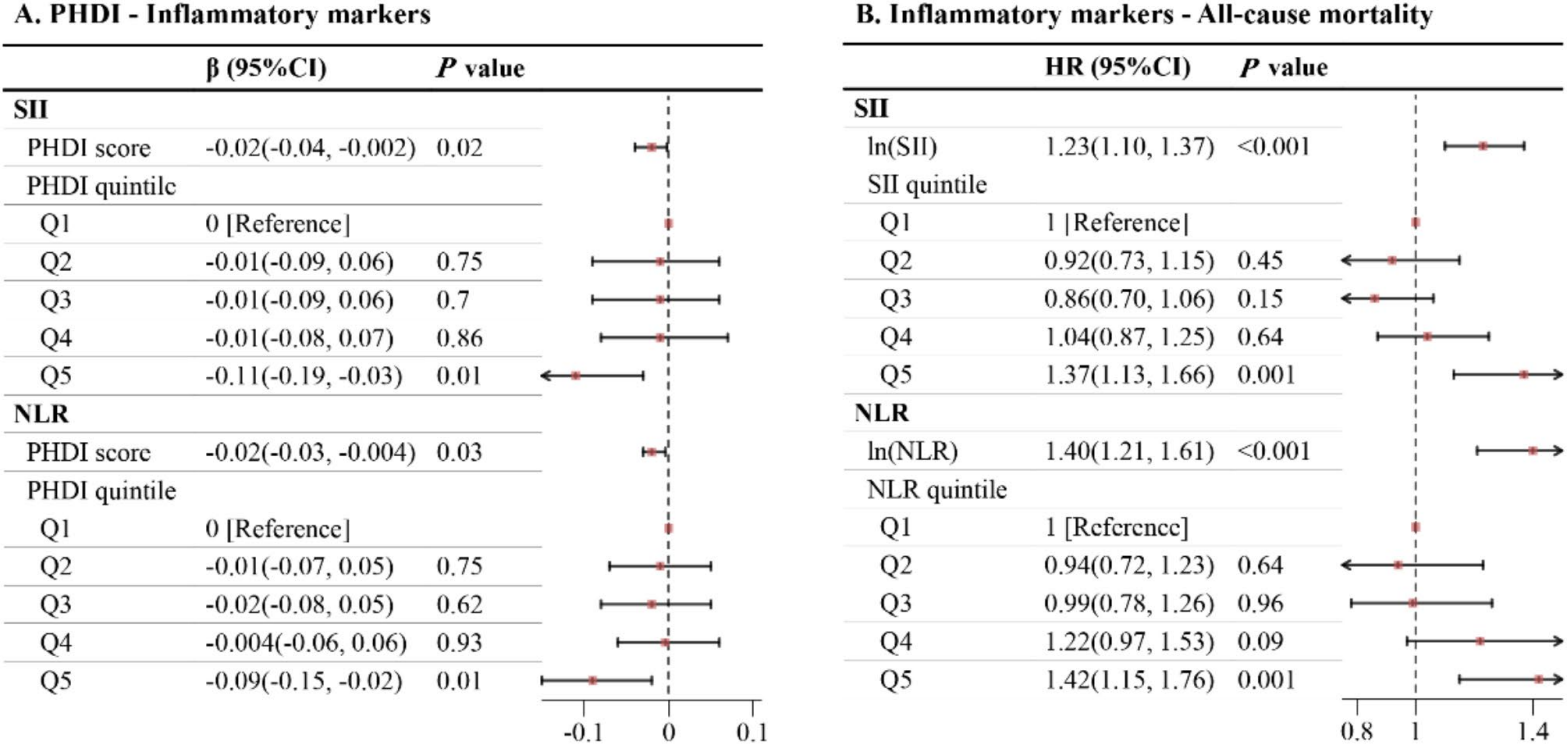
Association of PHDI with inflammatory markers and all-cause mortality among US cancer survivors. Note: **(A)** Association between PHDI and inflammatory markers among US cancer survivors. **(B)** Association between inflammatory markers and all-cause mortality among US cancer survivors. PHDI score was entered as a continuous variable per 10-point increase. The model was adjusted for age, sex, race/ethnicity, marital status, educational level, PIR, physical activity, alcohol use, smoke, BMI, energy, baseline year, hypertension, DM, and CVD. Abbreviations: BMI, body mass index; CI, Confidence interval; CVD, cardiovascular disease; DM, diabetes mellitus; NLR, neutrophil-to-lymphocyte ratio; PHDI, Planetary Health Diet Index; PIR, poverty income ratio; Q, quintile; SII, systemic immune-inflammation index

**Fig. 4:**
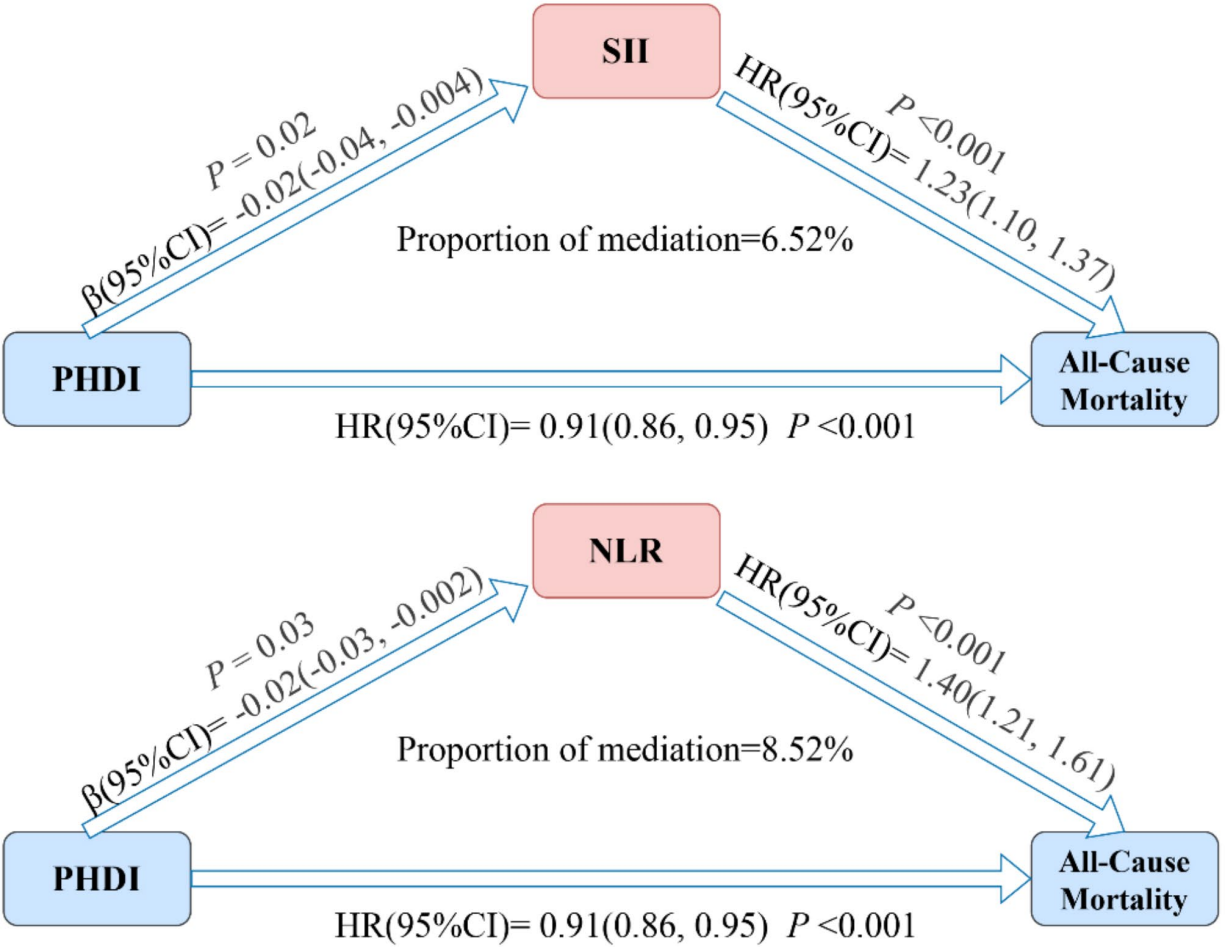
Mediation analysis of SII and NLR in the association between PHDI and all-cause mortality among US cancer survivors. The model was adjusted for age, sex, race/ethnicity, marital status, educational level, PIR, physical activity, alcohol use, smoke, BMI, energy, baseline year, hypertension, DM, and CVD. Abbreviations: BMI, body mass index; CVD, cardiovascular disease; DM, diabetes mellitus; NLR, neutrophil-to-lymphocyte ratio; PHDI, Planetary Health Diet Index; SII, systemic immune-inflammation index; PIR, poverty income ratio; HR, Hazard Ratio; CI, Confidence interval

Figure 3A demonstrates an inverse correlation between PHDI and both SII and NLR, while Fig. 3B shows a positive association of SII and NLR with all-cause mortality. The mediation analysis results presented in Fig. 4 indicate that the mediating proportions of SII and NLR are 6.52% and 8.52%, respectively. The mediating effects of SII and NLR between PHDI and all-cause mortality are detailed in Supplementary Table 5.

### Subgroup and sensitivity analyses

Subgroup analysis results indicate that no significant interactions between PHDI and all-cause mortality occurred in various subgroups (P for interaction > 0.05; Supplementary Table 6). As shown in Supplementary Table 7, excluding participants who passed away within two years does not alter the correlation between PHDI and all-cause, cancer, or non-cancer mortality, confirming the stability of these relationships. Supplementary Table 8 shows that after multiple imputations for missing covariates, the highest PHDI quintile was associated with a 35% lower risk of cancer mortality compared to the lowest quintile (HR, 0.65; 95% CI, 0.45–0.93), while other results remained stable.

## Discussion

The findings from this extensive, long-term prospective cohort study, with a median follow-up period surpassing a decade, demonstrated that an increase in PHDI was associated with a reduction in all-cause, cancer, and non-cancer mortality among cancer survivors. Additionally, PHDI showed a significant linear association with both all-cause mortality and non-cancer mortality among cancer survivors. It exhibited a significant inverse correlation with both SII and NLR, which in turn, were positively correlated with all-cause mortality. Mediation analysis indicated that SII and NLR accounted for 6.52% and 8.52% of the mediating effect between PHDI and all-cause mortality, respectively.

Most of the research has delved into the influence of individual dietary components, such as vegetables [25], nuts [26], multivitamins [27], and calcium [28], on cancer survivor mortality. However, focusing on single nutrients may not accurately capture their effects due to potential nutrient interactions and synergistic impacts, underscoring the importance of analyzing overall dietary patterns. Previous studies have investigated various dietary patterns in relation to cancer survivor prognosis. For example, Chen et al. [29] observed that adherence to the Mediterranean diet lowered mortality in breast cancer survivors, while Schwedhelm et al. [30] reported a significant association between following a prudent/healthy diet and reduced overall mortality in cancer survivors. PHD is a plant-based diet that uniquely incorporates environmental sustainability, distinguishing it from other dietary patterns. Attention to the PHD has increased in recent years in response to growing public health concerns about environmental issues. Studies of different cohorts, such as the Singapore Chinese Health Study [15], the Malmö Diet and Cancer Study [31], and the Nurses’ Health Study [16], reported that PHD adherence correlated with reduced all-cause and cancer mortality. However, few studies have specifically examined the correlation between PHD adherence and mortality in cancer survivors. In this investigation, we assessed PHD adherence by using the PHDI, a method whose reliability and feasibility have been validated in previous NHANES studies [10, 11, 32]. Our findings suggest that PHD may reduce mortality in cancer survivors, consistent with other dietary patterns [29, 30]. This indicates that PHD is both a beneficial dietary pattern for cancer prognosis and a sustainable choice for environmental health.

In the analysis of the association between PHDI components and all-cause mortality, whole fruits, non-starchy vegetables, nuts and seeds, unsaturated oils, saturated oils and trans fats, added sugar and fruit juice were significant. After excluding these six significant components, the association between the adjusted PHDI score and all-cause mortality was no longer statistically significant, indicating that their effects on all-cause mortality appear to be greater than those of the remaining components. Further analysis by cancer type revealed significant negative associations between PHDI and all-cause mortality in survivors of gynecological, male urological, and hematological cancers, with weaker associations in other cancer types, possibly due to differences in cancer treatments, environmental influences, cancer relapse, and small sample sizes. For a detailed discussion of these factors, please refer to the Supplementary Discussion. Future research is required to further clarify their specific impacts, allowing for a more comprehensive and accurate evaluation of the relationship between PHDI and mortality across different cancer types. Additionally, subgroup analyses indicated no significant interaction between PHDI and all-cause mortality across variables such as age, sex, and educational attainment, suggesting the findings can be generalized to US cancer populations with different characteristics.

Our study demonstrates a negative correlation between the PHDI and both the SII and NLR, suggesting that the PHD may reduce inflammatory levels in the body. The PHD focuses on plant-based foods, particularly a high intake of vegetables, fruits, whole grains, nuts, and legumes. Natural polyphenols, especially flavonoids in fruits and vegetables, inhibit enzymes involved in arachidonic acid metabolism, thus reducing pro-inflammatory mediators like prostaglandins and leukotrienes, while also modulating gene expression to lower pro-inflammatory transcription [33]. Resveratrol, another polyphenol, scavenges reactive oxygen species, inhibits cyclooxygenase, and activates anti-inflammatory pathways such as Sirt1 [34]. Furthermore, dietary fiber from whole grains reduces inflammation by altering gut pH and permeability [35, 36]. Antioxidants in nuts neutralize reactive oxygen species, lower oxidative stress, and inhibit NF-κB expression, leading to decreased production of pro-inflammatory cytokines [37]. Additionally, soy isoflavones and peptides target the NF-κB pathway, reducing inflammatory markers like interleukins and TNF-α [38].

In addition, our study reveals a significant positive association between SII, NLR, and all-cause mortality in cancer survivors. This indicates that SII and NLR can be valuable prognostic indicators for cancer survivors, aligning with previous studies [21, 39]. Elevated SII and NLR are associated with systemic inflammatory states that may facilitate cancer progression and recurrence, thereby increasing mortality risk. We also found that SII and NLR mediated the relationship between PHDI and all-cause mortality, suggesting that PHD may mitigate mortality risk for cancer survivors by improving inflammatory status. However, other mechanisms underlying this relationship remain poorly understood, warranting further research into how PHD influences cancer survivor health through additional biological pathways.

Our study has several limitations. First, as dietary information and cancer diagnoses were self-reported, recall bias could not be entirely ruled out. Second, despite adjusting for numerous confounding factors, some unmeasured confounders may remain, such as cancer stages, treatments, and relapse [8, 40]. Due to the lack of relevant data in NHANES, we are currently unable to investigate these factors. Additionally, the study population primarily consisted of American cancer survivors, limiting the generalizability of the results; further validation in different populations and regions is needed. While acknowledging these limitations, our study represents the first large-scale cohort investigation into the association between PHDI and mortality among cancer survivors, as well as the mediating roles of SII and NLR between PHDI and all-cause mortality. The findings provide valuable evidence for developing dietary recommendations for cancer survivors.

## Conclusions

This study underscores the potential of the PHD in reducing all-cause, cancer, and non-cancer mortality among cancer survivors and reveals the mediating role of inflammatory markers (SII and NLR) between PHDI and all-cause mortality. Adoption of this dietary pattern is of critical importance, as it not only enhances cancer prognosis but also aligns with the planet’s ecological carrying capacity.

## Electronic supplementary material

Below is the link to the electronic supplementary material.

Supplementary Material 1

## Data Availability

The datasets used and/or analyzed during the current study are available from the corresponding author on reasonable request.
