# Supplementary material for: Planetary health diet index and mortality among US cancer survivors: mediating roles of systemic immune-inflammation index and neutrophil-to-lymphocyte ratio
Source: Nutr J. 2025 Feb 22;24:28. doi: 10.1186/s12937-025-01097-6 (PMC11846200; doi:10.1186/s12937-025-01097-6)
Supplement: Supplementary file 1 — Supplementary Material 1 [file 12937_2025_1097_MOESM1_ESM.docx]

**Supplementary Materials Files:**

**Supplementary Text**

Supplementary Method. Cancer types.

Supplementary Method. Definition and categorization of covariates.

Supplementary Discussion. Detailed explanation on why the associations between PHDI and all-cause mortality are weaker in some cancer types.

**Supplementary Figure**

Supplementary Figure 1. Directed acyclic graph for the association between PHDI and all-cause mortality.

**Supplementary Table**

Supplementary Table 1. The Planetary Health Diet Index components and criteria for scoring.

Supplementary Table 2. Association of PHDI component score with all-cause, cancer, and noncancer mortality among US cancer survivors.

Supplementary Table 3. Association of adjusted PHDI score with all-cause, cancer, and noncancer mortality among US cancer survivors.

Supplementary Table 4. Association of PHDI with all-cause, cancer, and noncancer mortality in survivors with various cancer types.

Supplementary Table 5. The mediating effects of SII and NLR in the association between PHDI and all-cause mortality among US cancer survivors.

Supplementary Table 6. Association of PHDI with all-cause, cancer, and noncancer mortality among US cancer survivors in different subgroups.

Supplementary Table 7. Association of PHDI with all-cause, cancer, and noncancer mortality among US cancer survivors after excluding participants who died within 24 months.

Supplementary Table 8. Association of PHDI with all-cause, cancer, and noncancer mortality among US cancer survivors with multiple imputation analysis.

**Supplementary Method. Cancer types.**

Cancer types were classified into nine categories: gynecologic tumors (breast, cervical, ovarian, uterine), urologic tumors in males (prostate, testicular), head and neck tumors (laryngeal/tracheal, oral/tongue/lip, thyroid), respiratory system tumors (lung, laryngeal/tracheal), gastrointestinal tumors (colorectal, esophageal, gallbladder, hepatocellular, pancreatic, rectal, gastric), urologic tumors (bladder, renal cell), skin cancers (melanoma, non-melanoma), hematologic tumors (leukemia, lymphoma, other blood cancers), and other cancers.

**Supplementary Method. Definition and categorization of covariates.**

Age was categorized into two groups: younger than 65 years and 65 years or older. Self-reported race/ethnicity included Mexican American, non-Hispanic Black, non-Hispanic White, other Hispanic, or other. Marital status was classified as married, never married, living with a partner, or other (including widowed, divorced, or separated). Educational attainment was divided into three groups: less than high school, high school or equivalent, and above high school. Physical activity was categorized into three groups: inactive (0 minutes per week), insufficiently active (0 to <150 minutes per week), and active (≥150 minutes per week). Alcohol consumption was self-reported and classified as follows: never (fewer than 12 lifetime drinks), former (12 or more drinks in the past year but none in the past year, or none in the past year but 12 or more drinks in the lifetime), mild (up to 1 drink per day for women, up to 2 drinks per day for men), moderate (up to 2 drinks per day for women, up to 3 drinks per day for men), or heavy (3 or more drinks per day for women, 4 or more drinks per day for men). Smoking status was categorized into three groups: never (fewer than 100 lifetime cigarettes), former (100 or more lifetime cigarettes and quit), and now (100 or more lifetime cigarettes and currently smoking). Hypertension was diagnosed with a systolic blood pressure of 140 mmHg or higher or a diastolic blood pressure of 90 mmHg or higher. The diagnostic criteria for diabetes included a physician’s diagnosis, glycated hemoglobin (HbA1c) level of 6.5% or higher, a fasting blood glucose level of 7.0 mmol/L or higher, a random or 2-hour oral glucose tolerance test (OGTT) blood glucose level of 11.1 mmol/L or higher, or the use of diabetes medication/insulin. A history of cardiovascular disease was self-reported and included previous diagnoses of heart failure, coronary heart disease, angina, heart attack, or stroke. Body mass index (BMI) was calculated as weight in kilograms divided by the square of height in meters. Dietary energy intake data were collected via 24-hour dietary recall interviews conducted at the Mobile Examination Center (MEC).

**Supplementary Discussion. Detailed explanation on why the associations between PHDI and all-cause mortality are weaker in some cancer types.**

The relatively weak correlation between PHDI and all-cause mortality in certain cancer types can be attributed to several factors:

Firstly, treatment strategies influence the effect of PHDI. Different cancers are treated with distinct approaches. Intensive treatments such as high-dose chemotherapy and radiotherapy may lead to malnutrition or cachexia, thus reducing the long-term impact of PHDI on prognosis. For example, lung cancer survivors undergoing radiotherapy often experience weight loss(1), which could obscure the protective effects of PHDI. Similarly, gastrointestinal cancer survivors may experience restricted nutritional intake due to surgical resection(2), further influencing PHDI’s long-term effects. Additionally, cancers with poor prognoses, such as pancreatic ductal adenocarcinoma and hepatocellular carcinoma, have very low 5-year survival rates(3,4), so even with a high PHDI, mortality may not be significantly reduced. In contrast, cancers like prostate and breast cancer, with higher survival rates(5,6), may benefit more from PHDI’s long-term effects, thus reducing mortality.

Secondly, environmental factors also play a significant role. Some cancers are primarily driven by environmental factors rather than diet, such as skin cancer, which is closely related to ultraviolet radiation(7).

Thirdly, cancer relapse can significantly influence mortality. For example, a study by Balboa-Barreiro et al. found that the 5-year colorectal cancer-related mortality rate was 3.8% for patients who were recurrence-free one year post-surgery, compared to 33.6% for those with recurrence(8).

Finally, sample size affects the statistical power of results. In cancer types with smaller sample sizes, hazard ratio (HR) estimates may be unstable, leading to weaker statistical significance.

In conclusion, the weaker correlation between PHDI and all-cause mortality in some cancer types can be explained by factors such as treatment approaches, environmental factors, cancer relapse, and small sample size.

References:

1. Kiss N, Isenring E, Gough K, Krishnasamy M. The prevalence of weight loss during (chemo)radiotherapy treatment for lung cancer and associated patient- and treatment-related factors. Clin Nutr. 2014 Dec;33(6):1074–80.

2. Deftereos I, Yeung JMC, Arslan J, Carter VM, Isenring E, Kiss N, et al. Assessment of Nutritional Status and Nutrition Impact Symptoms in Patients Undergoing Resection for Upper Gastrointestinal Cancer: Results from the Multi-Centre NOURISH Point Prevalence Study. Nutrients. 2021 Sep 24;13(10):3349.

3. Stoffel EM, Brand RE, Goggins M. Pancreatic Cancer: Changing Epidemiology and New Approaches to Risk Assessment, Early Detection, and Prevention. Gastroenterology. 2023 Apr;164(5):752–65.

4. Chidambaranathan-Reghupaty S, Fisher PB, Sarkar D. Hepatocellular carcinoma (HCC): Epidemiology, etiology and molecular classification. Adv Cancer Res. 2021;149:1–61.

5. Tan EH, Burn E, Barclay NL, Delmestri A, Man WY, Golozar A, et al. Incidence, Prevalence, and Survival of Prostate Cancer in the UK. JAMA Netw Open. 2024 Sep 3;7(9):e2434622.

6. Barclay NL, Burn E, Delmestri A, Duarte-Salles T, Golozar A, Man WY, et al. Trends in incidence, prevalence, and survival of breast cancer in the United Kingdom from 2000 to 2021. Sci Rep. 2024 Aug 17;14(1):19069.

7. Perez M, Abisaad JA, Rojas KD, Marchetti MA, Jaimes N. Skin cancer: Primary, secondary, and tertiary prevention. Part I. J Am Acad Dermatol. 2022 Aug;87(2):255–68.

8. Balboa-Barreiro V, Pértega-Díaz S, García-Rodríguez T, González-Martín C, Pardeiro-Pértega R, Yáñez-González-Dopeso L, et al. Colorectal cancer recurrence and its impact on survival after curative surgery: An analysis based on multistate models. Dig Liver Dis. 2024 Jul;56(7):1229–36.

**Supplementary Figure 1. Directed acyclic graph for the association between PHDI and all-cause mortality.**


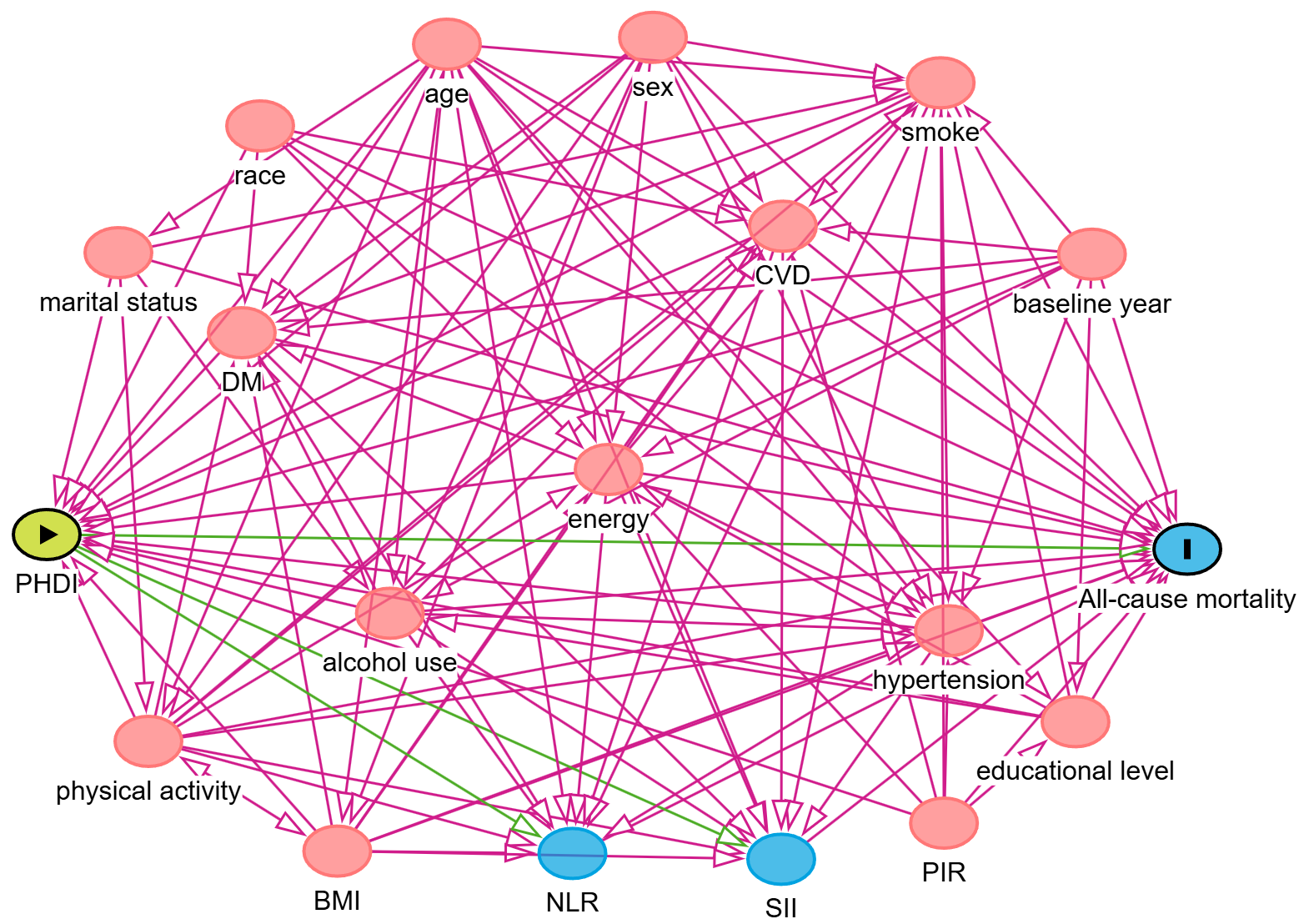


Abbreviations: BMI, body mass index; CVD, cardiovascular disease; DM, diabetes mellitus; NLR, neutrophil-to-lymphocyte ratio; PHDI, Planetary Health Diet Index; PIR, poverty income ratio; SII, systemic immune-inflammation index.

**Supplementary Table 1. The Planetary Health Diet Index components and criteria for scoring.**

| **Dietary component** | **Min score (0 points)** | **Max score (10 points)** | **Weight in total score** |
| --- | --- | --- | --- |
| **Adequacy components** |  |  |  |
| Whole grains | 0 g/d | ≥75 g/d (female)  ≥90 g/d (male) | 1 |
| Whole fruits^a^ | 0 g/d | ≥200 g/d | 1 |
| Nonstarchy vegetables | 0 g/d | ≥300 g/d | 1 |
| Nuts and seeds | 0 g/d | ≥50 g/d | 1 |
| Legumes |  |  |  |
| Nonsoy legumes | 0 g/d | 100 g/d | 0.5 |
| Soybean/ soy foods | 0 g/d | 50 g/d | 0.5 |
| Unsaturated oils^b^ | 0% of total energy intake | ≥10% of total energy intake | 1 |
| **Moderation components** |  |  |  |
| Starchy vegetables | ≥200 g/d | ≤50 g/d | 1 |
| Dairy | ≥4.08 serving-equivalents | ≤1.02 serving-equivalents | 1 |
| Red and processed meat | ≥300 g/d | ≤14 g/d | 1 |
| Poultry | ≥58 g/d | ≤29 g/d | 1 |
| Eggs | ≥120 g/d | ≤12 g/d | 1 |
| Fish | ≥50 g/d | ≤15 g/d | 1 |
| Saturated oils and trans fats | ≥21% of total energy intake | ≤3.5% of total energy intake | 1 |
| Added sugar and fruit juice | ≥25% of total energy intake | ≤5% of total energy intake | 1 |

^a^. The fruits exclude fruit juice.

^b^. To calculate the score for the legumes component, both the nonsoy and soy subcomponents were weighted equally at 0.5 each.

**Supplementary Table 2. Association of PHDI component score with all-cause, cancer, and noncancer mortality among US cancer survivors.**

|  | **All-Cause Mortality** |  | **Cancer Mortality** |  | **Noncancer Mortality** |  |
| --- | --- | --- | --- | --- | --- | --- |
| **PHDI component score** | **HR(95%CI)** | ***P* value** | **HR(95%CI)** | ***P* value** | **HR(95%CI)** | ***P* value** |
| **Adequacy components** |  |  |  |  |  |  |
| Whole grains score | 1.05(0.86, 1.27) | 0.64 | 0.87(0.60, 1.26) | 0.45 | 1.15(0.93, 1.41) | 0.19 |
| Whole fruits score | 0.81(0.67, 0.98) | 0.03 | 0.82(0.60, 1.12) | 0.21 | 0.79(0.63, 1.00) | 0.05 |
| Nonstarchy vegetables score | 0.76(0.60, 0.95) | 0.01 | 0.93(0.60, 1.45) | 0.76 | 0.69(0.53, 0.91) | 0.01 |
| Nuts and seeds score | 0.72(0.57, 0.92) | 0.01 | 0.84(0.52, 1.35) | 0.47 | 0.66(0.48, 0.90) | 0.01 |
| Legumes score | 0.74(0.45, 1.20) | 0.22 | 0.73(0.35, 1.53) | 0.41 | 0.74(0.41, 1.33) | 0.31 |
| Unsaturated oils score | 0.72(0.58, 0.90) | 0.005 | 0.70(0.49, 0.98) | 0.04 | 0.74(0.53, 1.03) | 0.08 |
| **Moderation components** |  |  |  |  |  |  |
| Starchy vegetables score | 0.98(0.78, 1.23) | 0.87 | 0.88(0.57, 1.35) | 0.54 | 1.05(0.81, 1.36) | 0.72 |
| Dairy score | 0.95(0.74, 1.21) | 0.65 | 1.26(0.73, 2.19) | 0.41 | 0.83(0.63, 1.09) | 0.17 |
| Red and processed meat score | 0.95(0.65, 1.39) | 0.80 | 1.25(0.69, 2.25) | 0.46 | 0.84(0.51, 1.39) | 0.50 |
| Poultry score | 1.04(0.87, 1.25) | 0.64 | 1.00(0.72, 1.41) | 0.98 | 1.06(0.88, 1.27) | 0.53 |
| Eggs score | 0.89(0.66, 1.19) | 0.43 | 0.74(0.39, 1.41) | 0.36 | 0.98(0.66, 1.46) | 0.93 |
| Fish score | 0.93(0.77, 1.12) | 0.44 | 0.85(0.61, 1.18) | 0.33 | 0.96(0.76, 1.21) | 0.73 |
| Saturated oils and trans fats score | 0.69(0.53, 0.91) | 0.01 | 0.80(0.49, 1.31) | 0.37 | 0.65(0.48, 0.89) | 0.01 |
| Added sugar and fruit juice score | 0.79(0.62, 1.00) | 0.046 | 0.86(0.53, 1.40) | 0.55 | 0.73(0.55, 0.98) | 0.04 |

Abbreviations: BMI, body mass index; CVD, cardiovascular disease; DM, diabetes mellitus; PHDI, Planetary Health Diet Index; PIR, poverty income ratio; HR, Hazard Ratio; CI, Confidence interval. The model was adjusted for age, sex, race/ethnicity, marital status, educational level, PIR, physical activity, alcohol use, smoke, BMI, energy, baseline year, hypertension, DM, and CVD. All PHDI component score was entered as a continuous variable per 10-point increase.

**Supplementary Table 3. Association of adjusted PHDI score with all-cause, cancer, and noncancer mortality among US cancer survivors.**

|  | **All-Cause Mortality** |  | **Cancer Mortality** |  | **Noncancer Mortality** |  |
| --- | --- | --- | --- | --- | --- | --- |
| **Adjusted PHDl score** | **HR(95%CI)** | ***P* value** | **HR(95%CI)** | ***P* value** | **HR(95%CI)** | ***P* value** |
| No whole grains score^a^ | 0.88(0.84, 0.93) | <0.001 | 0.90(0.81, 0.99) | 0.04 | 0.87(0.82, 0.93) | <0.001 |
| No whole fruits score^a^ | 0.90(0.85, 0.95) | <0.001 | 0.90(0.82, 0.99) | 0.03 | 0.90(0.84, 0.97) | 0.005 |
| No nonstarchy vegetables score^a^ | 0.91(0.86, 0.96) | <0.001 | 0.90(0.81, 0.99) | 0.03 | 0.91(0.85, 0.97) | 0.004 |
| No nuts and seeds score^a^ | 0.90(0.86, 0.95) | <0.001 | 0.90(0.82, 0.98) | 0.02 | 0.91(0.85, 0.97) | 0.003 |
| No legumes score^a^ | 0.90(0.86, 0.95) | <0.001 | 0.91(0.83, 0.99) | 0.03 | 0.90(0.85, 0.96) | <0.001 |
| No unsaturated oils score^a^ | 0.91(0.86, 0.96) | <0.001 | 0.91(0.83, 1.00) | 0.05 | 0.91(0.85, 0.96) | 0.002 |
| No starchy vegetables score^a^ | 0.90(0.86, 0.95) | <0.001 | 0.91(0.83, 0.99) | 0.03 | 0.90(0.84, 0.95) | <0.001 |
| No dairy score^a^ | 0.91(0.86, 0.95) | <0.001 | 0.90(0.82, 0.98) | 0.02 | 0.91(0.86, 0.96) | 0.002 |
| No red and processed meat score^a^ | 0.91(0.86, 0.95) | <0.001 | 0.90(0.82, 0.98) | 0.02 | 0.91(0.86, 0.96) | 0.001 |
| No poultry score^a^ | 0.90(0.86, 0.95) | <0.001 | 0.90(0.83, 0.98) | 0.02 | 0.90(0.84, 0.96) | <0.001 |
| No eggs score^a^ | 0.90(0.86, 0.95) | <0.001 | 0.91(0.84, 0.99) | 0.04 | 0.90(0.85, 0.96) | <0.001 |
| No fish score^a^ | 0.91(0.87, 0.96) | <0.001 | 0.90(0.84, 1.00) | 0.05 | 0.91(0.86, 0.96) | 0.001 |
| No saturated oils and trans fats score^a^ | 0.91(0.86, 0.96) | <0.001 | 0.90(0.82, 0.99) | 0.03 | 0.91(0.85, 0.97) | 0.003 |
| No added sugar and fruit juice score^a^ | 0.91(0.86, 0.96) | <0.001 | 0.90(0.82, 0.99) | 0.04 | 0.91(0.86, 0.97) | 0.003 |
| PHDI without eight components^b^ | 0.85(0.80, 0.91) | <0.001 | 0.89(0.79, 1.01) | 0.06 | 0.83(0.77, 0.90) | <0.001 |
| PHDI without six components^c^ | 0.97(0.89, 1.07) | 0.55 | 0.91(0.77, 1.08) | 0.29 | 1.00(0.90, 1.12) | 0.93 |

^a^. Calculated by subtracting each component score from the total PHDI score. For example, the “No red and processed meat score” represents the PHDI excluding the red and processed meat component.

^b^. The PHDI score without eight components (whole grains, legumes, starchy vegetables, dairy, red and processed meat, poultry, eggs, and fish) is calculated by subtracting the scores of these components from the total PHDI score.

^c^. The PHDI score without six components (whole fruits, nonstarchy vegetables, nuts and seeds, unsaturated oils, saturated oils and trans fats, and added sugar and fruit juice score) is calculated by subtracting the scores of these components from the total PHDI score.

Abbreviations: BMI, body mass index; CVD, cardiovascular disease; DM, diabetes mellitus; PHDI, Planetary Health Diet Index; PIR, poverty income ratio; HR, Hazard Ratio; CI, Confidence interval. The model was adjusted for age, sex, race/ethnicity, marital status, educational level, PIR, physical activity, alcohol use, smoke, BMI, energy, baseline year, hypertension, DM, and CVD. All adjusted PHDI score was entered as a continuous variable per 10 points increase.

**Supplementary Table 4. Association of PHDI score with all-cause, cancer, and noncancer mortality in survivors with various cancer types.**

|  |  | **All-Cause Mortality** |  | **Cancer Mortality** |  | **Noncancer Mortality** |  |
| --- | --- | --- | --- | --- | --- | --- | --- |
| **Cancer type**^a^ | **Number** | **HR(95%CI)** | ***P* value** | **HR(95%CI)** | ***P* value** | **HR(95%CI)** | ***P* value** |
| **Gynecologic Tumors** | 969 | 0.80(0.69, 0.93) | 0.003 | 1.12(0.94, 1.32) | 0.20 | 0.67(0.56, 0.81) | <0.001 |
| **Urologic Tumors (Male)** | 577 | 0.89(0.81, 0.98) | 0.02 | 0.71(0.60, 0.85) | <0.001 | 0.98(0.88, 1.09) | 0.72 |
| **Head and Neck Tumors** | 116 | 3.32(0.57, 19.33) | 0.18 | NA^b^ | NA | NA | NA |
| **Respiratory System Tumors** | 103 | 0.72(0.44, 1.18) | 0.20 | 0.72(0.26, 2.02) | 0.54 | 0.84(0.26, 2.66) | 0.77 |
| **Gastrointestinal Tumors** | 306 | 0.91(0.79, 1.04) | 0.15 | 0.91(0.76, 1.10) | 0.32 | 0.83(0.66, 1.05) | 0.12 |
| **Urologic Tumors** | 150 | 0.77(0.53, 1.12) | 0.18 | 1.08(0.81, 1.44) | 0.60 | 0.58(0.36, 0.93) | 0.02 |
| **Skin Cancers** | 1,140 | 0.97(0.88, 1.07) | 0.53 | 1.02(0.83, 1.25) | 0.86 | 0.95(0.85, 1.07) | 0.43 |
| **Hematologic Tumors** | 119 | 0.38(0.31, 0.46) | <0.001 | 0.27(0.22, 0.33) | <0.001 | NA | NA |
| **Other Cancers** | 238 | 0.85(0.67, 1.08) | 0.18 | 0.88(0.61, 1.29) | 0.52 | 0.90(0.63, 1.28) | 0.56 |

Abbreviations: BMI, body mass index; CVD, cardiovascular disease; DM, diabetes mellitus; PHDI, Planetary Health Diet Index; PIR, poverty income ratio; HR, Hazard Ratio; CI, Confidence interval. PHDI score was entered as a continuous variable per 10-point increase. The model was adjusted for age, sex, race/ethnicity, marital status, educational level, PIR, physical activity, alcohol use, smoke, BMI, energy, baseline year, hypertension, DM, and CVD.

^a^. Cancer types were classified into nine categories: gynecologic tumors (breast, cervical, ovarian, uterine), urologic tumors in males (prostate, testicular), head and neck tumors (laryngeal/tracheal, oral/tongue/lip, thyroid), respiratory system tumors (lung, laryngeal/tracheal), gastrointestinal tumors (colorectal, esophageal, gallbladder, hepatocellular, pancreatic, rectal, gastric), urologic tumors (bladder, renal cell), skin cancers (melanoma, non-melanoma), hematologic tumors (leukemia, lymphoma, other blood cancers), and other cancers.

^b^. NA means that HR and its confidence interval or P for interaction could not be calculated due to the small sample size or number of people with outcome events.

**Supplementary Table 5. The mediating effects of SII and NLR in the association between PHDI and all-cause mortality among US cancer survivors.**

|  | **Total effect(95CI%)** | ***P* value** | **Direct effect(95CI%)** | ***P* value** | **Indirect effect(95CI%)** | ***P* value** | **PM** | ***P* value** |
| --- | --- | --- | --- | --- | --- | --- | --- | --- |
| SII | 17.49(13.76, 26.88) | <0.001 | 16.35(12.90, 26.17) | <0.001 | 1.14(0.16, 2.01) | 0.01 | 6.52% | 0.01 |
| NLR | 16.78(14.10, 26.98) | <0.001 | 15.35(12.44, 25.85) | <0.001 | 1.43(0.23, 2.26) | 0.02 | 8.52% | 0.02 |

Abbreviations: CI, Confidence interval; NLR, neutrophil-to-lymphocyte ratio; PHDI, Planetary Health Diet Index; PM, Proportion mediation; SII, systemic immune-inflammation index.

**Supplementary Table 6. Association of PHDI with all-cause, cancer, and noncancer mortality among US cancer survivors in different subgroups.**

|  | **All-Cause Mortality** |  | **Cancer Mortality** |  | **Noncancer Mortality** |  |
| --- | --- | --- | --- | --- | --- | --- |
|  | **HR (95%CI)** | ***P* value** | **HR (95%CI)** | ***P* value** | **HR (95%CI)** | ***P* value** |
| Age group |  | 0.31* |  | 0.60* |  | 0.12* |
| 20-64 | 0.96(0.82, 1.12) | 0.58 | 1.01(0.82, 1.25) | 0.94 | 0.91(0.72, 1.16) | 0.46 |
| ≥65 | 0.89(0.85, 0.94) | <0.001 | 0.87(0.77, 0.97) | 0.01 | 0.91(0.85, 0.96) | 0.001 |
| Sex |  | 0.79* |  | 0.01* |  | 0.21* |
| Female | 0.89(0.80, 0.98) | 0.02 | 1.09(0.94, 1.26) | 0.25 | 0.81(0.72, 0.91) | <0.001 |
| Male | 0.91(0.84, 0.98) | 0.01 | 0.80(0.70, 0.91) | <0.001 | 0.96(0.89, 1.05) | 0.38 |
| Race/ethnicity |  | 0.46* |  | NA* |  | NA* |
| Mexican American | 0.88(0.66, 1.17) | 0.37 | 1.01(0.59, 1.75) | 0.96 | 0.73(0.48, 1.10) | 0.13 |
| Non-Hispanic Black | 0.95(0.81, 1.11) | 0.50 | 0.92(0.73, 1.17) | 0.50 | 0.98(0.81, 1.18) | 0.81 |
| Non-Hispanic White | 0.91(0.86, 0.96) | <0.001 | 0.90(0.82, 0.99) | 0.04 | 0.91(0.86, 0.97) | 0.004 |
| Other Hispanic | 0.75(0.33, 1.71) | 0.49 | 0.84(0.57, 1.25) | 0.39 | NA | NA |
| Other Race | 2.34(0.98, 5.57) | 0.06 | NA | NA | NA | NA |
| Marital status |  | 0.22* |  | NA* |  | NA* |
| Married | 0.89(0.83, 0.95) | <0.001 | 0.89(0.78, 1.01) | 0.08 | 0.89(0.81, 0.96) | 0.005 |
| Never married | 1.34(0.85, 2.11) | 0.20 | NA | NA | 1.70(0.88, 3.29) | 0.11 |
| Living with partner | NA | <0.001 | NA | NA | NA | NA |
| Other | 0.94(0.87, 1.02) | 0.13 | 0.98(0.85, 1.12) | 0.74 | 0.93(0.83, 1.03) | 0.18 |
| Education attainment |  | 0.92* |  | 0.38* |  | 0.76* |
| Less than high school | 0.93(0.83, 1.05) | 0.26 | 1.10(0.91, 1.34) | 0.33 | 0.88(0.77, 1.00) | 0.06 |
| High school or equivalent | 0.93(0.83, 1.04) | 0.19 | 0.88(0.70, 1.11) | 0.29 | 0.95(0.83, 1.08) | 0.43 |
| Above high school | 0.88(0.80, 0.96) | 0.004 | 0.86(0.75, 0.98) | 0.03 | 0.89(0.81, 0.98) | 0.01 |
| Physical activity |  | 0.23* |  | 0.22* |  | 0.62* |
| None (inactive) | 0.95(0.87, 1.04) | 0.27 | 0.98(0.84, 1.14) | 0.78 | 0.94(0.85, 1.04) | 0.25 |
| 0 to <150 (insufficiently active) | 0.91(0.82, 1.02) | 0.09 | 0.97(0.79, 1.20) | 0.78 | 0.90(0.79, 1.02) | 0.11 |
| ≥150 (active) | 0.88(0.79, 0.98) | 0.02 | 0.82(0.69, 0.99) | 0.04 | 0.92(0.81, 1.04) | 0.16 |
| Alcohol use |  | 0.30* |  | 0.61* |  | 0.33* |
| Never | 0.83(0.70, 0.98) | 0.03 | 0.79(0.57, 1.09) | 0.16 | 0.91(0.78, 1.05) | 0.20 |
| Former | 0.92(0.83, 1.01) | 0.08 | 0.87(0.73, 1.05) | 0.14 | 0.93(0.83, 1.04) | 0.21 |
| Mild | 0.96(0.88, 1.06) | 0.42 | 0.97(0.82, 1.15) | 0.76 | 0.96(0.87, 1.05) | 0.38 |
| Moderate | 0.83(0.65, 1.05) | 0.12 | 0.89(0.66, 1.21) | 0.46 | 0.67(0.45, 0.99) | 0.04 |
| Heavy | 0.87(0.71, 1.06) | 0.16 | 1.01(0.77, 1.32) | 0.96 | 0.77(0.52, 1.15) | 0.20 |
| Smoke |  | 0.31* |  | 0.20* |  | 0.63* |
| Never | 0.84(0.77, 0.92) | <0.001 | 0.82(0.66, 1.01) | 0.07 | 0.85(0.77, 0.94) | 0.001 |
| Former | 0.91(0.84, 0.97) | 0.01 | 0.88(0.79, 0.99) | 0.03 | 0.91(0.84, 0.99) | 0.04 |
| Now | 1.04(0.90, 1.22) | 0.58 | 1.12(0.83, 1.52) | 0.44 | 1.00(0.79, 1.26) | 0.99 |
| Hypertension |  | 0.68* |  | 0.49* |  | 0.81* |
| No | 0.92(0.83, 1.02) | 0.11 | 0.94(0.80, 1.11) | 0.48 | 0.91(0.80, 1.04) | 0.17 |
| Yes | 0.89(0.84, 0.94) | <0.001 | 0.87(0.79, 0.96) | 0.01 | 0.90(0.84, 0.96) | 0.002 |
| DM |  | 0.65* |  | 0.21* |  | 0.24* |
| No | 0.90(0.85, 0.95) | <0.001 | 0.93(0.84, 1.03) | 0.15 | 0.88(0.82, 0.95) | <0.001 |
| Yes | 0.90(0.80, 1.01) | 0.07 | 0.82(0.68, 1.00) | 0.05 | 0.93(0.82, 1.05) | 0.22 |
| CVD |  | 0.18* |  | 0.81* |  | 0.23* |
| No | 0.88(0.83, 0.94) | <0.001 | 0.89(0.80, 0.99) | 0.04 | 0.88(0.82, 0.95) | <0.001 |
| Yes | 0.93(0.85, 1.02) | 0.13 | 0.92(0.80, 1.07) | 0.28 | 0.92(0.82, 1.04) | 0.18 |

Abbreviations: BMI, body mass index; CVD, cardiovascular disease; DM, diabetes mellitus; PHDI, Planetary Health Diet Index; PIR, poverty income ratio; HR, Hazard Ratio; CI, Confidence interval.

Note: The model was adjusted for age, sex, race/ethnicity, marital status, educational level, PIR, physical activity, alcohol use, smoke, BMI, energy, baseline year, hypertension, DM, and CVD (exclude the subgroup variable). PHDI score was entered as a continuous variable per 10-point increase. NA means that HR and its confidence interval or P for interaction could not be calculated due to the small sample size or number of people with outcome events.

^*^. *P* for interaction.

**Supplementary Table 7. Association of PHDI with all-cause, cancer, and noncancer mortality among US cancer survivors after excluding participants who died within 24 months.**

|  | **Model 1^a^** |  | **Model 2^b^** |  | **Model 3^c^** |  |
| --- | --- | --- | --- | --- | --- | --- |
| **Mortality Outcome** | **HR(95%CI)** | ***P* value** | **HR(95%CI)** | ***P* value** | **HR(95%CI)** | ***P* value** |
| **All-Cause Mortality** |  |  |  |  |  |  |
| PHDI quintile |  |  |  |  |  |  |
| Q1 | 1[Reference] |  | 1[Reference] |  | 1[Reference] |  |
| Q2 | 0.85(0.68, 1.05) | 0.14 | 0.94(0.77, 1.15) | 0.55 | 0.91(0.74, 1.12) | 0.38 |
| Q3 | 0.89(0.73, 1.10) | 0.28 | 1.07(0.86, 1.34) | 0.52 | 1.06(0.85, 1.32) | 0.62 |
| Q4 | 0.72(0.57, 0.90) | 0.005 | 0.89(0.71, 1.12) | 0.34 | 0.86(0.68, 1.09) | 0.22 |
| Q5 | 0.55(0.42, 0.71) | <0.001 | 0.73(0.57, 0.94) | 0.01 | 0.72(0.57, 0.92) | 0.01 |
| Trend test |  | <0.001 |  | 0.01 |  | 0.01 |
| **Cancer Mortality** |  |  |  |  |  |  |
| PHDI quintile |  |  |  |  |  |  |
| Q1 | 1[Reference] |  | 1[Reference] |  | 1[Reference] |  |
| Q2 | 0.81(0.55, 1.21) | 0.31 | 0.94(0.63, 1.42) | 0.78 | 0.96(0.64, 1.45) | 0.85 |
| Q3 | 0.99(0.67, 1.48) | 0.97 | 1.28(0.88, 1.87) | 0.20 | 1.29(0.88, 1.89) | 0.20 |
| Q4 | 0.74(0.48, 1.14) | 0.17 | 0.96(0.63, 1.46) | 0.85 | 0.97(0.64, 1.49) | 0.90 |
| Q5 | 0.66(0.43, 1.03) | 0.07 | 0.98(0.63, 1.53) | 0.94 | 0.99(0.63, 1.54) | 0.96 |
| Trend test |  | 0.06 |  | 0.97 |  | 0.98 |
| **Noncancer Mortality** |  |  |  |  |  |  |
| PHDI quintile |  |  |  |  |  |  |
| Q1 | 1[Reference] |  | 1[Reference] |  | 1[Reference] |  |
| Q2 | 0.86(0.66, 1.13) | 0.28 | 0.94(0.72, 1.23) | 0.66 | 0.90(0.67, 1.19) | 0.45 |
| Q3 | 0.86(0.65, 1.13) | 0.29 | 1.00(0.74, 1.37) | 0.98 | 0.98(0.72, 1.33) | 0.89 |
| Q4 | 0.71(0.53, 0.95) | 0.02 | 0.87(0.63, 1.21) | 0.41 | 0.83(0.59, 1.15) | 0.26 |
| Q5 | 0.51(0.38, 0.68) | <0.001 | 0.66(0.49, 0.87) | 0.004 | 0.64(0.48, 0.85) | 0.002 |
| Trend test |  | <0.001 |  | 0.005 |  | 0.003 |

Abbreviations: BMI, body mass index; CVD, cardiovascular disease; DM, diabetes mellitus; PHDI, Planetary Health Diet Index; PIR, poverty income ratio; Q, quintile; HR, Hazard Ratio; CI, Confidence interval.

^a^. Adjusted for age.

^b^. Adjusted for age, sex, race/ethnicity, marital status, educational level, PIR, physical activity, alcohol use, smoke, BMI, energy, and baseline year.

^c^. Adjusted for age, sex, race/ethnicity, marital status, educational level, PIR, physical activity, alcohol use, smoke, BMI, energy, baseline year, hypertension, DM, and CVD.

**Supplementary Table 8. Association of PHDI with all-cause, cancer, and noncancer mortality among US cancer survivors with multiple imputation analysis.**

|  | **All-Cause Mortality** |  | **Cancer Mortality** |  | **Noncancer Mortality** |  |
| --- | --- | --- | --- | --- | --- | --- |
|  | **HR(95%CI)** | ***P* value** | **HR(95%CI)** | ***P* value** | **HR(95%CI)** | ***P* value** |
| **Before MI** |  |  |  |  |  |  |
| PHDI quintile |  |  |  |  |  |  |
| Q1 | 1[Reference] |  | 1[Reference] |  | 1[Reference] |  |
| Q2 | 0.85(0.69, 1.04) | 0.11 | 0.85(0.57, 1.25) | 0.40 | 0.85(0.66, 1.09) | 0.20 |
| Q3 | 0.95(0.77, 1.17) | 0.62 | 1.05(0.74, 1.50) | 0.78 | 0.91(0.70, 1.19) | 0.49 |
| Q4 | 0.79(0.63, 0.98) | 0.03 | 0.85(0.57, 1.27) | 0.42 | 0.77(0.57, 1.03) | 0.08 |
| Q5 | 0.67(0.53, 0.83) | <0.001 | 0.79(0.52, 1.20) | 0.27 | 0.62(0.47, 0.81) | <0.001 |
| **After MI** |  |  |  |  |  |  |
| PHDI quintile |  |  |  |  |  |  |
| Q1 | 1[Reference] |  | 1[Reference] |  | 1[Reference] |  |
| Q2 | 0.88(0.74, 1.07) | 0.20 | 0.81(0.58, 1.12) | 0.20 | 0.93(0.74, 1.17) | 0.54 |
| Q3 | 1.02(0.85, 1.22) | 0.87 | 1.05(0.77, 1.43) | 0.76 | 1.01(0.80, 1.26) | 0.96 |
| Q4 | 0.77(0.64, 0.94) | 0.01 | 0.76(0.54, 1.06) | 0.11 | 0.79(0.62, 0.99) | 0.04 |
| Q5 | 0.69(0.56, 0.84) | <0.001 | 0.65(0.45, 0.93) | 0.02 | 0.70(0.54, 0.9) | 0.01 |

Abbreviations: BMI, body mass index; CVD, cardiovascular disease; DM, diabetes mellitus; PHDI, Planetary Health Diet Index; PIR, poverty income ratio; Q, quintile; MI, multiple imputation; HR, Hazard Ratio; CI, Confidence interval. The model was adjusted for age, sex, race/ethnicity, marital status, educational level, PIR, physical activity, alcohol use, smoke, BMI, energy, baseline year, hypertension, DM, and CVD.
